# Types of deviation and review criteria in pretreatment central quality control of tumor bed boost in medulloblastoma—an analysis of the German Radiotherapy Quality Control Panel in the SIOP PNET5 MB trial

**DOI:** 10.1007/s00066-021-01822-0

**Published:** 2021-08-05

**Authors:** Stefan Dietzsch, Annett Braesigk, Clemens Seidel, Julia Remmele, Ralf Kitzing, Tina Schlender, Martin Mynarek, Dirk Geismar, Karolina Jablonska, Rudolf Schwarz, Montserrat Pazos, Damien C. Weber, Silke Frick, Kristin Gurtner, Christiane Matuschek, Semi Ben Harrabi, Albrecht Glück, Victor Lewitzki, Karin Dieckmann, Martin Benesch, Nicolas U. Gerber, Denise Obrecht, Stefan Rutkowski, Beate Timmermann, Rolf-Dieter Kortmann

**Affiliations:** 1 Department of Radiation Oncology, University of Leipzig Medical Center, Stephanstr. 9a, 04103 Leipzig, Germany; 2 Clinic for Particle Therapy, West German Proton Therapy Centre, University of Essen, Essen, Germany; 3 Departement of Pediatric Hematology and Oncology, University Medical Center Hamburg-Eppendorf, Hamburg, Germany; 4 Faculty of Medicine, Department of Radiation Oncology, University of Cologne, Cologne, Germany; 5 Department of Radiation Oncology, University Medical Center Hamburg-Eppendorf, Hamburg, Germany; 6 Department of Radiotherapy and Radiation Oncology, Ludwig Maximilian University Munich, Munich, Germany; 7 Center for Protontherapy, Paul Scherrer Institute, Villigen, Switzerland; 8 Department of Radiotherapy and Radiation Oncology, Hospital Bremen Mitte, Bremen, Germany; 9 Department of Radiotherapy and Radiation Oncology, Faculty of Medicine and University Hospital, Carl Gustav Carus, Technical University Dresden, Dresden, Germany; 10 Department of Radiation Oncology, Medical Faculty Heinrich Heine University Duesseldorf, Duesseldorf, Germany; 11 Department of Radiation Oncology and Radiotherapy, Heidelberg University Hospital, Heidelberg, Germany; 12 Radiation Oncology, Munich-Schwabing Municipal Hospital, Munich, Germany; 13 Department of Radiotherapy, University of Wuerzburg, Wuerzburg, Germany; 14 Department of Radiotherapy, Medical University of Vienna, Vienna, Austria; 15 Division of Pediatric Hematology/Oncology, Department of Pediatrics and Adolescent Medicine, Medical University of Graz, Graz, Austria; 16 Department of Oncology, University Children’s Hospital, Zurich, Switzerland

**Keywords:** Brain tumor, Pediatric, Focal radiotherapy, Quality assurance, Individual case review

## Abstract

**Purpose:**

In Germany, Austria, and Switzerland, pretreatment radiotherapy quality control (RT-QC) for tumor bed boost (TB) in non-metastatic medulloblastoma (MB) was not mandatory but was recommended for patients enrolled in the SIOP PNET5 MB trial between 2014 and 2018. This individual case review (ICR) analysis aimed to evaluate types of deviations in the initial plan proposals and develop uniform review criteria for TB boost.

**Patients and methods:**

A total of 78 patients were registered in this trial, of whom a subgroup of 65 patients were available for evaluation of the TB treatment plans. Dose uniformity was evaluated according to the definitions of the protocol. Additional RT-QC criteria for standardized review of target contours were elaborated and data evaluated accordingly.

**Results:**

Of 65 initial TB plan proposals, 27 (41.5%) revealed deviations of target volume delineation. Deviations according to the dose uniformity criteria were present in 14 (21.5%) TB plans. In 25 (38.5%) cases a modification of the RT plan was recommended. Rejection of the TB plans was rather related to unacceptable target volume delineation than to insufficient dose uniformity.

**Conclusion:**

In this analysis of pretreatment RT-QC, protocol deviations were present in a high proportion of initial TB plan proposals. These findings emphasize the importance of pretreatment RT-QC in clinical trials for MB. Based on these data, a proposal for RT-QC criteria for tumor bed boost in non-metastatic MB was developed.

**Supplementary Information:**

The online version of this article (10.1007/s00066-021-01822-0) contains supplementary material, which is available to authorized users.

## Introduction

Postoperative craniospinal irradiation (CSI) with boost delivery is a cornerstone of treatment of medulloblastomas (MB) [1]. In standard-risk patients, the boost volume has changed from the posterior fossa (PF) to the tumor bed (TB) [2]. For PF boost, retrospective reports showed that inadequate treatment volumes were applied in a substantial proportion of patients [3–5]. A retrospective analysis of the French *Medulloblastome-Société Française d’Oncologie Pédiatrique* 1998 (M-SFOP 98) trial revealed adequate target volume and dose distribution of tumor bed boost [6]. Experiences with focal radiotherapy in other brain tumors using similar target volume concepts, however, showed significant rates of radiotherapy (RT) protocol deviations [7–10].

The International Society of Pediatric Oncology Peripheral Primitive Neuroectodermal Tumor 5 MB trial (SIOP PNET5 MB, ClinicalTrials.gov identifier: NCT02066220) included a TB boost with a reduced clinical target volume (CTV) margin of 1.0 cm compared to previously published trials [6, 11, 12]. Protocol-compliant application of the TB boost was required to draw conclusions on the CTV margin out of the relapse patterns inside the posterior fossa. This report presents first data of pretreatment radiotherapy quality control (RT-QC) of TB boost in patients enrolled to the trial in Germany, Switzerland, and Austria between 2014 and 2018. The analysis details the types of deviations observed in the initial plan proposals submitted by local departments of radiation oncology and describes uniform review criteria for future MB studies. The pattern of relapse will be a subject of the final analysis of the protocol.

## Patients and methods

In the SIOP PNET5 MB trial, pretreatment central RT-QC of CSI plans was mandatory. RT-QC was organized on a national basis. Data on patients enrolled in Germany, Switzerland, and Austria, including details on treatment schedule, treatment techniques, workflow of RT-QC, results of RT-QC of CSI plans, and the German radiotherapy control panel, have been published elsewhere [13]. In the first years of trial recruitment, transfer and central review of Digital Imaging and Communications in Medicine (DICOM)-RT data with Magnetic Resonance Imaging (MRI) fusion could not be ensured in all participating countries. Therefore, pretreatment RT-QC of TB boost was optional in the first protocol versions (version 10, February 21, 2013; and version 11, November 17, 2014). However, pretreatment RT-QC was recommended by the German radiotherapy control panel. According to the protocol, the national radiotherapy QC panels were responsible for determining the criteria by which TB boost treatment plans were evaluated. For dose uniformity, minor and major deviations were used prospectively, which were defined in consensus of the national RT coordinators according to International Commission on Radiation Units and Measurements (ICRU) 50/62 and which were also used for RT-QC of 3D conformal CSI plans from the beginning of the central review and incorporated into the protocol version 12 (June 29, 2017) [14]. The criteria are described in Table 1. This protocol version was initiated in Germany in November 2018 and did not include definitions of target volume deviations for TB boost. For target delineation, RT-QC criteria were adapted from the SIOP Ependymoma II protocol (ClinicalTrials.gov identifier: NCT02265770) for evaluation (Table 1). Furthermore, deviations were rated for clinical relevance and scored as “acceptable” or “unacceptable” as described elsewhere [13].

**Table 1:** Protocol definitions of RT parameters for quality control and additional definitions of minor and major deviations in target delineation used by the reference center

	Per protocol	Minor deviation	Major deviation
GTV	Resection cavity and/or residual tumor	Not defined	Not defined
CTV	GTV +1 cm except bone and tentorium	Not defined	Not defined
PTV	CTV +0.3 to 0.5 cm	Not defined	Not defined
*Dose uniformity*			
V95%	≥ 95%	≥ 90 to < 95%	< 90%
V107%	≤ 5%	> 5% to < 10%	≥ 10%
*Additional definitions of target delineation minor and major deviations*			
CTV	1.0 cm	< 1.0 cm PTV margin	Not defined
GTV	–	Encompass normal brain tissue	Not encompass MRI visible resection cavity/residual tumor

*GTV* gross tumor volume, *CTV* clinical target volume, *PTV* planning target volume, *V95* volume of PTV receiving ≥ 95% of the prescribed dose, *V107* volume of PTV receiving ≥ 107% of the prescribed dose

Details of the treatment recommendations of the protocol were presented and discussed with the local radiation oncologists at meetings of the *Arbeitsgemeinschaft pädiatrische Radioonkologie* (APRO; Working Group Pediatric Radiation Oncology). A benchmark case was not performed. Protocol deviations were communicated to the local radiation oncologists via telephone or email including illustrating screenshots.

Central plan analyses were performed using the treatment planning systems (TPS) of the reference center (RayStation, Raysearch Laboratories, Stockholm, Sweden). The original dose file of the local radiotherapy was imported and evaluated. A re-calculation was not done, only a re-sampling of dose–volume parameters was performed in case of modified volumes of interest. Use of the original MRI-CT co-registration matrix was established at the end of the observed period. Therefore, new image registrations with the cranial MRIs were performed in almost all cases, taking into account the uncertainties of co-registration differences between TPS of the local radiotherapy unit and the system of the reference center.

Associations between variables were examined using Fisher exact and χ^2^ tests. All statistical analyses were performed using the Statistical Package for Social Sciences (IBM SPSS statistics), version 24 (IBM, Armonk, NY, USA).

## Results

**Fig. 1:**
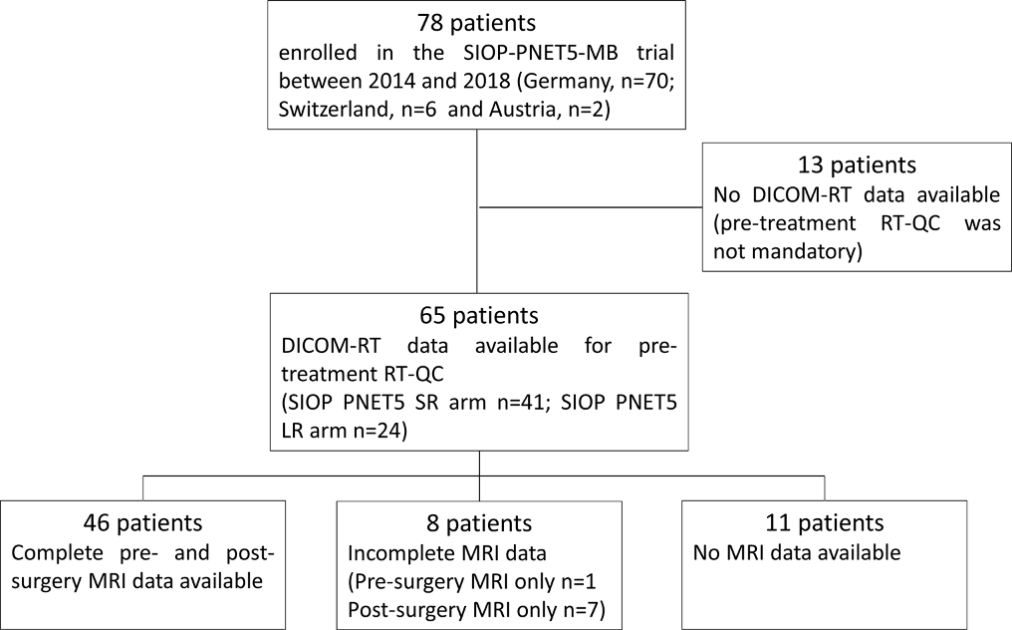
Consort diagram of the present analysis. *SIOP-PNET5-MB* The International Society of Pediatric Oncology Peripheral Primitive Neuroectodermal Tumor 5 Medulloblastoma trial, *DICOM* Digital Imaging and Communications in Medicine, *RT* radiotherapy, *QC* quality control, *MRI* magnetic resonance imaging, *SR* standard risk, *LR* low risk

Between September 2014 and December 2018, 78 patients (Germany, *n* = 70; Switzerland, *n* = 6, and Austria, *n* = 2) were enrolled in the SIOP-PNET5-MB trial. DICOM-RT data were unavailable in 13 (19%) patients; thus, 65 boost (81%) plans were analyzed. The majority (41/65; 63.1%) of the patients were treated according to the SIOP PNET5 SR arm (CSI 23.4 Gy + TB boost 30.6 Gy) and 24/65 patients (36.9%) according to the arm for patients with a low-risk biological profile (SIOP PNET5 LR arm, CSI 18.0 Gy + TB boost 36.0 Gy). Pre- and post-surgery MRIs were available for review in 46 (70.8%) patients, whereas 7 patients (10.8%) had only post-surgery MRI and 1 patient (1.5%) only pre-surgery MRI. In 11 patients (16.9%) MRI was not available for plan review (Fig. 1).

**Fig. 2:**
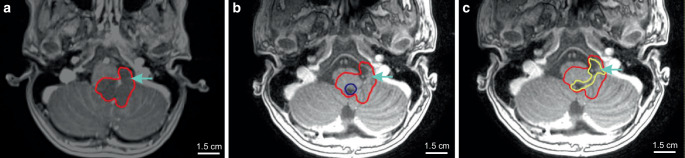
Example of a major/unacceptable deviation; **a** shows the pre-surgery MRI with the initial tumor volume (*red*); **b** shows the post-surgery MRI with the GTV_tumorbed_ (*blue*) of the local radiation oncologist, which does not encompass the MRI visible resection cavity/areas with initial tumor contact (initial tumor volume, *red*); **c** shows the reference GTV_tumorbed_ (*yellow*) and the initial tumor volume (*red*). The *green arrow* marks the region of deviation. *MRI* magnetic resonance imaging, *GTV* gross tumor volume

Target volume deviations occurred in 41.5% of cases (Supplementary Table 1). The most common reason for target volume deviation was incorrect clinical target volume (CTV) and/or planning target volume (PTV) margin (26.2%; Supplementary Figure 1). Delineation of resection cavity (gross tumor volume, GTV_tumorbed_) was incorrect in 20.0% of plans. Predominantly, GTV_tumorbed_ was too large and encompassed normal brain tissue. In 4 patients, GTV_tumorbed_ did not encompass the complete resection cavity (Fig. 2). In 14 patients without complete MRI data, no deviation of GTV_tumorbed_ was detected when reviewing the planning CT. However, a final judgement on correctness of the GTV_tumorbed_ was not possible in these patients.

In 5 of 53 (9.4%) cases with available post-surgery MRI, a striking discrepancy between the resection cavity on MRI, performed within 48 h after surgery, and the planning CT was found (Supplementary Figure 2). The interval between post-surgery MRI and planning CT was median 29 (range 18 to 35) days in these patients.

Dose uniformity deviations were found in 23.1% of plans and were predominantly minor (18.5%). The median V95 (volume of PTV receiving ≥ 95% of the prescribed dose) was 98.5 ± 3.3%. A V95 < 95% was observed in 14 (21.5%) of plans. In one plan (1.5%) the V107 (volume of PTV receiving ≥ 107% of the prescribed dose) was more than 5%. RT technique had no impact on the frequency of dose uniformity deviations (Supplementary Table 2; Fisher exact test, *p* = 0.509).

Overall, 34 plans (52.3%) did not reveal any deviation (Supplementary Table 3). In 9.2% of plans, deviations were considered acceptable and in 25 (38.5%) plans, modifications were recommended. In the cohort of plans with deviations (*n* = 31), no significant correlation between the defined minor and major deviations and the acceptance of plans was seen (unacceptable plans 71.4 vs. 83.3%; chi^2^ *p* = 0.483, Table 2). Moreover, there was no difference in the rate of unacceptable plans between patients with or without complete MRI data (37.0 vs. 42.1%; chi^2^ *p* = 0.875). However, 10 of the 14 patients with incomplete MRI data and no deviation of GTV_tumorbed_ detected in the planning CT were finally scored as acceptable with the remark of incomplete review.

**Table 2:** Relationship between types of deviation (target volume and/or dose uniformity) and overall result of quality control (acceptable/unacceptable)

	Per protocol	Acceptable deviation	Unacceptable deviation	Total
Correct target and dose	34	0	0	*34 (52.3%)*
Correct target but deviation dose uniformity	0	3 dose minor *n* = 2 dose major 0 = 1	1 dose major *n* = 1	*4 (6.2%)*
Deviation target but correct dose uniformity	0	2 target minor *n* = 1 target major *n* = 1	14 target minor *n* = 12 target major *n* = 2	*16 (24.6%)*
Deviation target and dose uniformity	0	1 dose minor *n* = 1 target minor *n* = 1	10 dose minor *n* = 9 dose major *n* = 1 target minor *n* = 9 target major *n* = 1	*11 (16.9%)*
Total	*34 (52.3%)*	*6 (9.2%)*	*25 (38.5%)*	*65 (100%)*

**Fig. 3:**
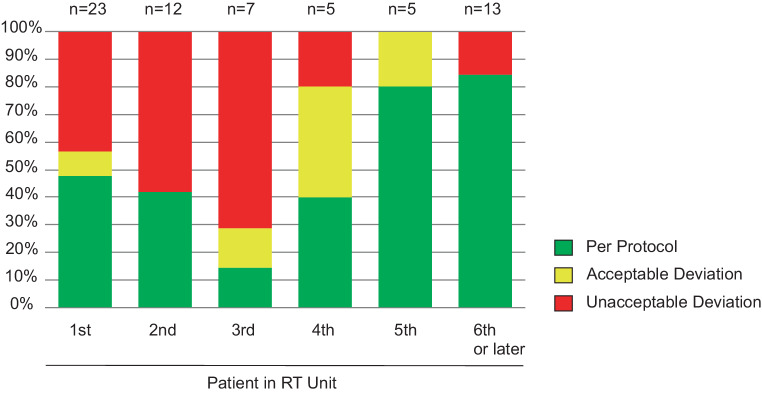
Influence of experience within the protocol on quality control result. *n* depicts number of patients who were evaluated with respect to the patients in radiotherapy unit (e.g., 23 patients were evaluated as the first patient in an unit)

There was no difference in the need for plan modifications between low-recruiting (≤ 4 patients) and high-recruiting (≥ 5 patients) radiotherapy units (42.3% vs. 35.9%; chi^2^ *p* = 0.461). Fig. 3 shows the impact of institutional experiences in treating SIOP PNET5 MB trial patients on the rate of unacceptable RT plans. Most of the unacceptable boost plans occurred in the first to third patient of each radiotherapy unit (22/25; 88%). The decline of the ratio was significant (first to third patient in unit 52.4% vs. fourth or later patient in unit 13.0%; *p* = 0.008). However, 3 cases of unacceptable plans were also observed in the second group (fouth or later patient in unit).

Table 2 summarizes the type of observed deviation and the need for plan modification. Only 1 plan with dose uniformity deviation alone required plan modification. In 14 of 25 (56%) cases with unacceptable deviations, only target volume deviations were present. Of these patients 2/14 (14.3%) had major deviations (GTV did not encompass the resection cavity), 1/14 (7.1%) minor deviation (GTV encompassed too much normal brain tissue), 10/14 (71.4%) minor deviations (incorrect CTV/PTV margin), and 1/14 (7.1%) minor deviation (both, i.e., GTV encompassed too much normal brain tissue and incorrect CTV/PTV margin). In 10/25 patients (40%), unacceptable plans showed both target volume and dose uniformity deviations.

## Discussion

The present study represents a large cohort of MB patients undergoing a pretreatment, fully digital, individual case review QC procedure in TB boost for MB.

### Observed deviations

We observed RT plan deviations in approximately 50% of cases and recommended plan modification in 38.5% of plans. To date, no comparable data on pretreatment RT-QC of 3D conformal TB boost in MB is available. Previous reports examined deviations of simulation-based PF boost and observed frequent inaccuracies. However, most of the deviations were considered minor or seemed to have no significant impact on event-free or overall survival [3, 5, 15, 16]. In contrast, RT-QC analysis in the SIOP United Kingdom Children’s Cancer Study Group (UKCCSG) PNET3 trial revealed a significantly higher PF recurrence rate in patients treated with PF-targeting deviation (34.4% vs. 16.3%; *p* = 0.043). However, no significant impact on overall survival (*p* = 0.4034) was observed [4].

Retrospective quality control of TB boosts in the FRENCH M‑SFOP 98 trial could not show any inadequacies of target definition and dose distribution. However, details and definitions of RT-QC criteria were not provided within that report [6].

The planning procedure of tumor bed boost in MB is comparable to local irradiation of other brain tumor entities. The target volumes consist of the tumor bed and/or residual tumor with an isotropic safety margin for subclinical disease of about 0.5 to 1.5 cm (CTV margin), which has to be adapted to anatomical borders. The CTV margin depends on the infiltrating potential of the tumor or special character of the tumor, e.g., growing of meningiomas along the meninges. Another safety margin is added for the setup error (PTV margin). In low-grade gliomas, a retrospective RT-QC analysis resulted in overall grades of minor and major deviation of 37% and 32%, respectively [7]. In malignant/atypical meningiomas, Coskun et al. observed major and minor deviations in 22% and 10% of reviewed plans, respectively [8]. The RT-QC of the CATNON intergroup trial included a retrospective individual case review for each first patient randomized per institution: 35.5% of the cases were evaluated as per protocol, 17.7% as acceptable variation, and 46.8% as unacceptable [10].

In our study, we also observed high rates of target volume deviations (41.5%). The requirement for boost plan modification was rather related to target deviations than to dose uniformity issues. High rates of target volume variations (71%) were also observed in the CATNON cohort, resulting in unacceptable variations in 27% of the cases [10]. Retrospective RT-QC of the EORTC 22033–26033 trial also revealed high rates of incorrect volume delineation (63% minor, 23% major deviations) [7]. A high inter-clinician variation in contouring of TB in MB was also shown in a planning exercise prior to the SIOP PNET4 trial [17]. These findings point out that target delineation is the most critical part of treatment planning, emphasizing the role of central review of target volumes. Moreover, a training program for all participating investigators (e.g., a benchmark case) with a final certificate would be desirable for further investigations.

In contrast to other studies, in our cohort dose uniformity deviations were less frequent and not necessarily combined with unacceptable plans. This might be explained by the relatively low total dose of 54 Gy in the SIOP PNET5 study as compared to the EORTC 22042–26042 and CATNON trials with 60 Gy or more applied nearby critical organs, such as the brainstem or optic pathway, frequently leading to dose reductions [8, 10].

### Role of MRI co-registration

Incorrect delineation of GTV_tumorbed_ was observed in 20% of the plans and was comparable to other reports [7, 10]. Co-registration with initial and post-surgery imaging improves the accuracy of target delineation and is now standard for contouring of the resection cavity [18, 19]. Central review was based on CT/MRI co-registration of pre- and post-surgery MRI in 70.8% of the patients; 12.3% of patients had incomplete MRI data (only pre- or post-surgery MRI available) and in 16.9% of patients, no MRI was available. Availability of MRI co-registration was higher than in the cohorts of the EORTC 22042–26042 (36.8% no MRI data) or CATNON trials (proper co-registration in 42.9% of patients) [7, 10]. No difference in correlation of unacceptable plans with or without complete MRI data could be revealed. This may partly be explained by the high rate of incorrect CTV/PTV margins (26.2%) leading to unacceptable plans evaluable without any MRI. On the other hand, in 2 cases, refusal of plans was based on discrepancies between the initial tumor volume and the resection cavity as delineated by the local radiation oncologist without analyzing the MRI co-registration during review. In contrast, the resection cavity was considered correct when reviewing only the planning CT in 14 patients. In these cases, an uncertainty of review remained and was commented in the reviewing report. In the majority of cases, the original co-registration matrix was not available and the reviewer performed a new co-registration. This procedure has to take into account the possibility of co-registration differences. However, these differences seem to be limited in the cranial region. A multi-institutional benchmark test for cranial CT/MR image registration revealed an uncertainty of approximately 2 mm [20]. In 5 patients, we detected a discrepancy between the resection cavity visible in the post-surgery MRI, performed within 48 h after surgery, and the planning CT. Geometrical changes in the resection cavity before RT have also been reported in other brain tumor entities, e.g., gliomas or brain metastases [21, 22]. This issue needs to be further examined in order to understand whether an additional MRI would be mandatory at the time of RT planning or even during CSI before planning and starting the TB boost.

### New RT-QC criteria

No definitions for target volume deviations of the TB boost were available within the SIOP PNET5 MB protocol. In principle, target delineation of boost volume is comparable to local radiotherapy of other brain tumor entities. However, no consensus on target delineation deviations exists. Definitions differ between available reports (Supplementary Table 4) [7, 8, 10]. We defined incomplete coverage of the resection cavity/residual tumor by the GTV as major deviation. An insufficient margin or inclusion of uninvolved normal brain tissue into the GTV was considered a minor deviation (Table 1). However, no correlation between the defined major deviations and unacceptance of plans on one hand and between minor deviations and acceptance of plans on the other hand was seen. We observed a high number of incorrect CTV margins defined as minor deviation, often leading to the recommendation for plan modification due to an expected increase in the risk of relapse. In 9 cases, the resection cavity was delineated too large and unnecessarily encompassed normal brain tissue without an anticipated increased risk of relapse. Nevertheless, plans were considered unacceptable when a larger volume of uninvolved normal tissue was included due to the potentially higher risk of toxicity. Moreover, creation of the PTV is a multistep approach (GTV→CTV→PTV) and deviations in every step can lead to cumulation or a mutual compensation of errors. For example, in one case, the addition of an incorrect GTV, which was too large, and an incorrectly too small CTV margin (5 mm) resulted in a nearly correct and acceptable CTV/PTV (Supplementary Figure 3).

Taking all these findings into account, it appears necessary to renew the criteria for tumor bed RT-QC. Therefore, we have proposed appropriate definitions helping to better display relevant findings in a structured way in the future (Table 3). According to our proposal for CSI, modification of the PTV should be recommended if the reference CTV is not covered [13]. Moreover, a cutoff for too large PTVs needs to be defined depending on the location of the RT volume and, accordingly, to the expected increase in the risk of late toxicity (e.g., 0.5 cm inside the posterior fossa). In case of two or more deviations (GTV, CTV margin, and/or PTV margin), the potential addition of variations or mutual compensation of errors has to be taken into account, too. Therefore, it is recommended to create a correct reference CTV/PTV for final decision on these cases.

**Table 3:** Proposal for the definition of acceptable versus unacceptable deviations

**Target volume delineation**		
**Step 1**		
	*Acceptable deviation* *(as a single deviation)*	*Unacceptable deviation*
GTV_tumorbed_ does not encompass MRI visible resection cavity/residual tumor	≤ setup error margin	> setup error margin
GTV_tumorbed_ encompasses uninvolved normal brain tissue	≤ 0.5 cm	> 0.5 cm
CTV margin	≥ 0.5 cm–≤ 1.5 cm	< 0.5 cm or > 1.5 cm
PTV margin	≥ 0.2 cm–≤ 1 cm	< 0.2 cm or > 1 cm
**Step 2** **→** **If more than 1 deviation is present in step 1, it is recommended to create a correct reference CTV/PTV for final solution according to the following criteria**		
	*Acceptable deviation*	*Unacceptable deviation*
CTV/PTV	Reference CTV not encompassed by CTV but by PTV or PTV larger than necessary (≤ 0.5 cm)	Reference CTV not encompassed by PTV or PTV substantially larger than necessary (> 0.5 cm)
**Dose uniformity**		
**V95%**	≥ 95 to < 98%	< 95%
**V107%**	> 5% to < 10%	≥ 10%

*MRI* magnetic resonance imaging, *GTV* gross tumor volume, *CTV* clinical target volume, *PTV* planning target volume, *V95* volume of PTV receiving ≥ 95% of the prescribed dose, *V107* volume of PTV receiving ≥ 107% of the prescribed dose

Regarding dose uniformity, we found appropriate dose coverage in most of the cases. Therefore, we propose using stricter dose uniformity constraints (V95% ≥ 98%), as is recommended by the ICRU report 83 for intensity-modulated RT techniques (IMRT) and as proposed for CSI when using high-precision techniques [13, 23]. The same constraints shall also be used for proton plans, respecting the recommendations of the ICRU 78 report [24].

### Impact of experience in treating SIOP PNET5 MB patients

In contrast to our experiences in RT-QC of CSI plans and the published results for atypical and malignant meningioma, we did not see any difference in the need for plan modifications when comparing low-recruiting and high-recruiting centers (≤ 4 patients 42.3% vs. ≥ 5 patients 35.9%) [8, 13]. However, the majority of unacceptable plans (88%) occurred in patients who were among the first 3 patients of each institution. However, in 3 cases, plan modification was also recommended at a later stage of study participation. This is in contrast to our experiences with RT-QC in CSI plans, where no unacceptable plan occurred in a fifth or later patient of the respective institution [13]. This might be explained by the highly standardized contouring and planning process for CSI, whereas focal RT of TB is highly individual for the patients. However, the number of reviewed and rejected plans at a later stage of study participation is too low to come to reliable conclusions.

### Limitations of the study

The most important limitation is the reduced clinical significance of the study results due to the rapidly evolving irradiation technique. The first protocol versions included only review criteria of Carrie et al. for simulation-based CSI planning [3]. Dose uniformity criteria of this analysis were established according to the recommendations of the ICRU50/62 for 3D conformal irradiation [14]. However, in most of the cases, high-precision techniques like IMRT or proton beam therapy were used. Therefore, the proposed new constraints are based on the stricter recommendations of the ICRU 83 report [23]. As RT techniques will continue to develop, review criteria will have to be adapted, e.g., robust planning in proton beam therapy.

Another weakness is the incomplete availability of MRI data and the original MRI co-registration matrix, which cause uncertainties in the evaluation of the GTV (tumor bed).

## Conclusion

Comparable to our experiences in RT-QC of CSI plans, our findings on TB boost emphasize the impact of central pretreatment RT-QC in at least the first 3 to 5 patients per institution to ensure protocol-compliant treatment planning for clinical trials in MB [13]. The development of standardized RT-QC criteria, as proposed by us, enables consistent plan review and thereby may be of help for other studies. An MRI at the time of CT-based treatment planning of RT could improve the precision of target volume delineation.

## Supplementary Information

Supplementary Table 1: Frequency of deviations of target volume delineation and dose uniformity

Supplementary Figure 1: Example from the case study with incorrect clinical target volume margin

Supplementary Figure 2: Example from the case study with change of resection cavity between post-surgery magnetic resonance imaging and planning computer tomography

Supplementary Table 2: Impact of radiotherapy techniques on frequency of dose uniformity deviations

Supplementary Table 3: Final result of quality control for the whole cohort (n=65) and divided due to availability of complete magnetic resonance imaging data for central review

Supplementary Table 4: Definitions of target volume deviations of other trials

Supplementary Figure 3: Example from the case study with two acceptable deviations and mutual compensation of these errors

## References

[1] Northcott PA, Robinson GW, Kratz CP (2019). Medulloblastoma. Nat Rev Dis Primers.

[2] Michalski JM, Janss A, Vezina G (2016). Results of COG ACNS0331: a phase III trial of involved-field radiotherapy (IFRT) and low dose craniospinal irradiation (LD-CSI) with chemotherapy in average-risk Medulloblastoma: a report from the children’s oncology group. Int J Radiat Oncol Biol Phys.

[3] Carrie C, Hoffstetter S, Gomez F (1999). Impact of targeting deviations on outcome in medulloblastoma: study of the French Society of Pediatric Oncology (SFOP). Int J Radiat Oncol Biol Phys.

[4] Taylor RE, Bailey CC, Robinson KJ (2004). Impact of radiotherapy parameters on outcome in the International Society of Paediatric Oncology/United Kingdom Children’s Cancer Study Group PNET-3 study of preradiotherapy chemotherapy for M0-M1 medulloblastoma. Int J Radiat Oncol Biol Phys.

[5] Donahue B, Marymont MAH, Kessel S (2012). Radiation therapy quality in CCG/POG intergroup 9961: implications for craniospinal irradiation and the posterior fossa boost in future medulloblastoma trials. Front Oncol.

[6] Carrie C, Muracciole X, Gomez F (2005). Conformal radiotherapy, reduced boost volume, hyperfractionated radiotherapy, and online quality control in standard-risk medulloblastoma without chemotherapy: results of the French M-SFOP 98 protocol. Int J Radiat Oncol Biol Phys.

[7] Fairchild A, Weber DC, Bar-Deroma R (2012). Quality assurance in the EORTC 22033-26033/CE5 phase III randomized trial for low grade glioma: the digital individual case review. Radiother Oncol.

[8] Coskun M, Straube W, Hurkmans CW (2013). Quality assurance of radiotherapy in the ongoing EORTC 22042-26042 trial for atypical and malignant meningioma: results from the dummy runs and prospective individual case Reviews. Radiat Oncol.

[9] Gondi V, Cui Y, Mehta MP (2015). Real-time pretreatment review limits unacceptable deviations on a cooperative group radiation therapy technique trial: quality assurance results of RTOG 0933. Int J Radiat Oncol Biol Phys.

[10] Abrunhosa-Branquinho AN, Bar-Deroma R, Collette S (2018). Radiotherapy quality assurance for the RTOG 0834/EORTC 26053-22054/NCIC CTG CEC.1/CATNON intergroup trial “concurrent and adjuvant temozolomide chemotherapy in newly diagnosed non-1p/19q deleted anaplastic glioma”: Individual case review analysis. Radiother Oncol.

[11] Paulino AC, Mazloom A, Teh BS (2011). Local control after craniospinal irradiation, intensity-modulated radiotherapy boost, and chemotherapy in childhood medulloblastoma. Cancer.

[12] Merchant TE, Kun LE, Krasin MJ (2008). Multi-institution prospective trial of reduced-dose craniospinal irradiation (23.4 Gy) followed by conformal posterior fossa (36 Gy) and primary site irradiation (55.8 Gy) and dose-intensive chemotherapy for average-risk medulloblastoma. Int J Radiat Oncol Biol Phys.

[13] Dietzsch S, Braesigk A, Seidel C (2020). Pretreatment central quality control for craniospinal irradiation in non-metastatic medulloblastoma : first experiences of the German radiotherapy quality control panel in the SIOP PNET5 MB trial. Strahlenther Onkol.

[14] The International Commission of Radiation units and Measurements (1993) ICRU Report 50: Prescribing, Recording and Reporting Photon Beam Therapy. Journal of the ICRU (Volume os-26 Issue 1, September 1993). 10.1093/jicru_os26.1.iii

[15] Miralbell R, Bleher A, Huguenin P (1997). Pediatric medulloblastoma: radiation treatment technique and patterns of failure. Int J Radiat Oncol Biol Phys.

[16] Miralbell R, Fitzgerald TJ, Laurie F (2006). Radiotherapy in pediatric medulloblastoma: quality assessment of Pediatric Oncology Group Trial 9031. Int J Radiat Oncol Biol Phys.

[17] Coles CE, Hoole ACF, Harden SV (2003). Quantitative assessment of inter-clinician variability of target volume delineation for medulloblastoma: quality assurance for the SIOP PNET 4 trial protocol. Radiother Oncol.

[18] Rosenman JG, Miller EP, Tracton G (1998). Image registration: an essential part of radiation therapy treatment planning. Int J Radiat Oncol Biol Phys.

[19] Cattaneo GM, Reni M, Rizzo G (2005). Target delineation in post-operative radiotherapy of brain gliomas: interobserver variability and impact of image registration of MR(pre-operative) images on treatment planning CT scans. Radiother Oncol.

[20] Ulin K, Urie MM, Cherlow JM (2010). Results of a multi-institutional benchmark test for cranial CT/MR image registration. Int J Radiat Oncol Biol Phys.

[21] Scharl S, Kirstein A, Kessel KA (2019). Volumenveränderungen der Resektionshöhlen nach Operation von Hirnmetastasen – Konsequenzen für die stereotaktische Strahlentherapie (Cavity volume changes after surgery of a brain metastasis-consequences for stereotactic radiation therapy). Strahlenther Onkol.

[22] Wee CW, Kim IH, Park C-K (2020). Interim tumor progression and volumetric changes of surgical cavities during the surgery-to-radiotherapy interval in anaplastic gliomas: implications for additional pre-radiotherapy magnetic resonance imaging. Cancer Res Treat.

[23] The International Commission on Radiation Units and Measurements (2010). Prescribing, recording, and reporting intensity-modulated photon-beam therapy (IMRT). J ICRU.

[24] International Commission on Radiation Units and Measurements (2007). ICRU report 78: prescribing, recording and reporting proton-beam therapy. J ICRU.

