# Supplementary material for: Types of deviation and review criteria in pretreatment central quality control of tumor bed boost in medulloblastoma—an analysis of the German Radiotherapy Quality Control Panel in the SIOP PNET5 MB trial
Source: Strahlenther Onkol. 2021 Aug 5;198(3):282–90. doi: 10.1007/s00066-021-01822-0 (PMC8863746; doi:10.1007/s00066-021-01822-0)
Supplement: Supplementary file 1 — Supplementary Table 1: Frequency of deviations of target volume delineation and dose uniformity [file 66_2021_1822_MOESM1_ESM.pdf]

	n	per protocol	minor deviation	major deviation	total not per protocol (minor or major)
**Target volume Boost[a)]**	65	38 (58.4%)	23 (35.4%)	4 (6.2%)	**27 (41.5%)**
**Resection cavity[a)]**					
**All patients**	65	52 (80%)	9 (13.8%)	4 (6.2%).	13 (20.0%)
**Pre/post-surgery MRI available**	46	38 (82.6%)	7 (15.2%)	1 (2.2%)	8 (17.4%)
**No or incomplete MRI data**	19	14 (73.8%)	2 (10.5%)	3 (15.7%)	5 (26.3%)
**CTV/PTV-margin[a)]**	65	48 (73.8%)	17 (26.2%)	Not defined	17 (26.2%)
**Dose uniformity Boost**	65	50 (76.9%)	12 (18.5%)	3 (4.6%)	**15 (23.1%)**
**V95% Boost**	65	51 (78.5%)	12 (18.5%)	2 (3.1%)	14 (21.5%)
**V107% Boost**	65	64 (98.5%)	0	1 (1.5%)	1 (1.5%)

Supplementary Table 1 Deviation of target volume delineation and dose uniformity.

[a)] For target volume delineation, no definition of major and minor deviation are available in the protocol. Quality control was done according definitions in table 1.

MRI – magnetic resonance imaging

CTV – clinical target volume

PTV – planning target volume

V95 - volume of PTV receiving ≥95% of the prescribed dose

V107 - volume of PTV receiving ≥107% of the prescribed dose

 Dietzsch S et al. Department for Radiation Oncology, University of Leipzig Medical Center, Leipzig, Germany. Email: stefan.dietzsch@medizin.uni-leipzig.de
