# Supplementary material for: Types of deviation and review criteria in pretreatment central quality control of tumor bed boost in medulloblastoma—an analysis of the German Radiotherapy Quality Control Panel in the SIOP PNET5 MB trial
Source: Strahlenther Onkol. 2021 Aug 5;198(3):282–90. doi: 10.1007/s00066-021-01822-0 (PMC8863746; doi:10.1007/s00066-021-01822-0)
Supplement: Supplementary file 2 — Supplementary Figure 1: Example from the case study with incorrect clinical target volume margin [file 66_2021_1822_MOESM2_ESM.pdf]

Supplementary Figure 1

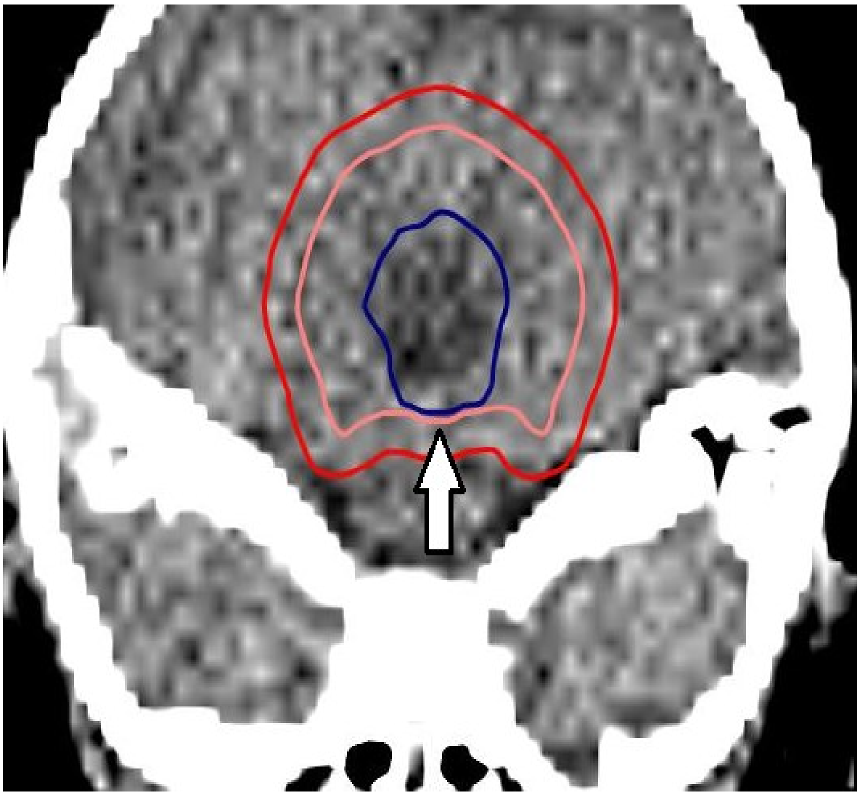

Example incorrect CTV margin – correct $GTV_{tumorbed}$ (blue) but incorrect CTV margin (pink) because exclusion of brainstem, which is not recommended in the protocol. Incorrect CTV margin was defined as minor deviation but often led to recommendation for plan modification (PTV – red)

Types of deviation and review criteria in pre-treatment central quality control of tumor bed boost in medulloblastoma – An analysis of the German Radiotherapy Quality Control Panel in the SIOP PNET5 MB trial. Strahlentherapie und Radioonkologie. Dietzsch S et al. Department for Radiation Oncology, University of Leipzig Medical Center, Leipzig, Germany. Email: stefan.dietzsch@medizin.uni-leipzig.de
