# Supplementary material for: Types of deviation and review criteria in pretreatment central quality control of tumor bed boost in medulloblastoma—an analysis of the German Radiotherapy Quality Control Panel in the SIOP PNET5 MB trial
Source: Strahlenther Onkol. 2021 Aug 5;198(3):282–90. doi: 10.1007/s00066-021-01822-0 (PMC8863746; doi:10.1007/s00066-021-01822-0)
Supplement: Supplementary file 3 — Supplementary Figure 2: Example from the case study with change of resection cavity between post-surgery magnetic resonance imaging and planning computer tomography [file 66_2021_1822_MOESM3_ESM.pdf]

Supplementary figure 2

A) post-surgery MRI

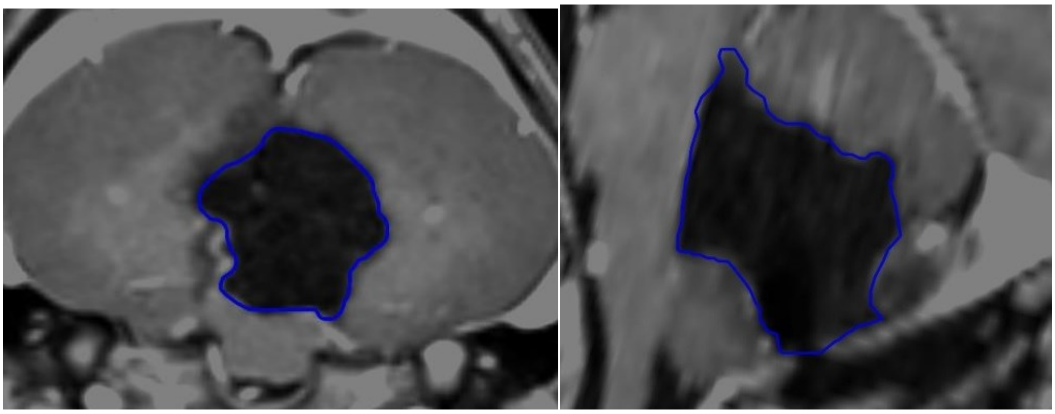

B) planning CT

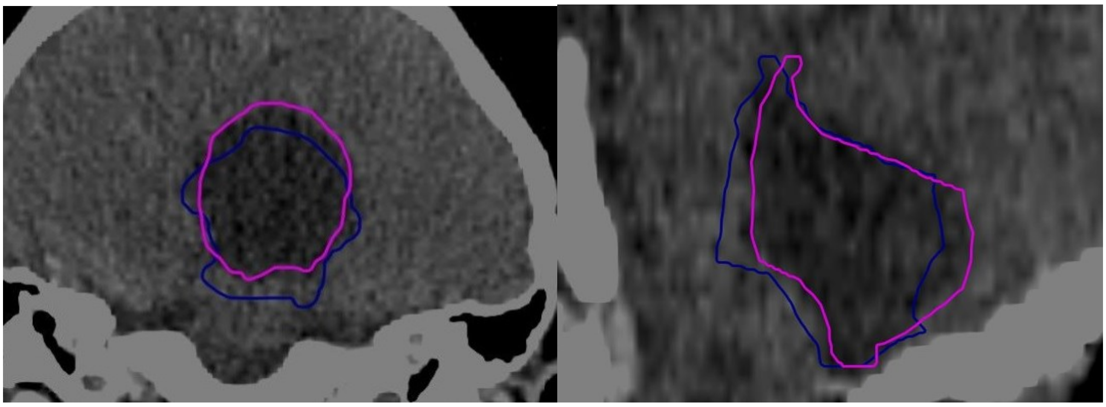

C) MRI before start of boost

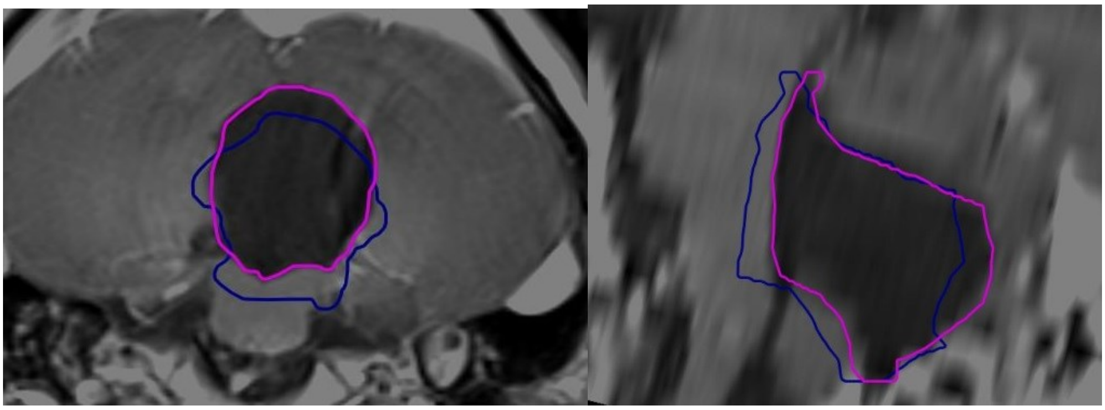

Example resection cavity change – initial $GTV_{tumorbed}$ (blue) was delineated according to the post-surgery MRI (A – performed at day 1 after surgery), because of discrepancy in the planning CT (B) MRI was repeated before start of boost (C) and the $GTV_{tumorbed}$ was modified (purple)

Types of deviation and review criteria in pre-treatment central quality control of tumor bed boost in medulloblastoma – An analysis of the German Radiotherapy Quality Control Panel in the SIOP PNET5 MB trial. Strahlentherapie und Radioonkologie. Dietzsch S et al. Department for Radiation Oncology, University of Leipzig Medical Center, Leipzig, Germany. Email: stefan.dietzsch@medizin.uni-leipzig.de
