# Supplementary material for: Types of deviation and review criteria in pretreatment central quality control of tumor bed boost in medulloblastoma—an analysis of the German Radiotherapy Quality Control Panel in the SIOP PNET5 MB trial
Source: Strahlenther Onkol. 2021 Aug 5;198(3):282–90. doi: 10.1007/s00066-021-01822-0 (PMC8863746; doi:10.1007/s00066-021-01822-0)
Supplement: Supplementary file 4 — Supplementary Table 2: Impact of radiotherapy techniques on frequency of dose uniformity deviations [file 66_2021_1822_MOESM4_ESM.pdf]

	n	per protocol	minor deviation	major deviation	total not per protocol (minor or major)
**3d conformal**	9	7 (77.8%)	2 (22.2%)	0	2 (22.2%)
**IMRT**	6	4 (66.7%)	2 (33.3%)	0	2 (33.3%)
**VMAT**	15	11 (73.3%)	2 (13.3%)	2 (13.3%)	4 (26.6%)
**Tomotherapy**	9	6 (66.7%)	2 (22.2%)	1 (11.1%)	3 (33.3%)
**Proton beam therapy**	26	22 (84.7%)	4 (15.4%)	0	4 (15.4%)
**Whole cohort**	65	50 (76.9%)	12 (18.5%)	3 (4.6%)	15 (23.1%)

Supplementary Table 2 Impact of radiotherapy techniques on frequency of dose uniformity deviations.

3d conformal – 3d conformal radiotherapy without intensity modulation
IMRT – intensity modulated radiotherapy with fixed gantry angles
VMAT – volumetric modulated arc therapy

Types of deviation and review criteria in pre-treatment central quality control of tumor bed boost in medulloblastoma – An analysis of the German Radiotherapy Quality Control Panel in the SIOP PNET5 MB trial. Strahlentherapie und Radioonkologie. Dietzsch S et al. Department for Radiation Oncology, University of Leipzig Medical Center, Leipzig, Germany. Email: stefan.dietzsch@medizin.uni-leipzig.de
