# Supplementary material for: Types of deviation and review criteria in pretreatment central quality control of tumor bed boost in medulloblastoma—an analysis of the German Radiotherapy Quality Control Panel in the SIOP PNET5 MB trial
Source: Strahlenther Onkol. 2021 Aug 5;198(3):282–90. doi: 10.1007/s00066-021-01822-0 (PMC8863746; doi:10.1007/s00066-021-01822-0)
Supplement: Supplementary file 5 — Supplementary Table 3: Final result of quality control for the whole cohort (n=65) and divided due to availability of complete magnetic resonance imaging data for central review [file 66_2021_1822_MOESM5_ESM.pdf]

	**Per protocol**	**Acceptable deviation**	**Unacceptable Deviation**
**All patients (n=65)**	34 (52.3%)	6 (9.2%)	25 (38.5%)
**Subgroups based on availability of MRI**			
**Pre/post-surgery MRI available (n=46)**	25 (54.3%)	4 (8.7%)	17 (37.0%)
**No or incomplete MRI (n=19)**	9 (47.4%)	2 (10.5%)	8 (42.1%)

Supplementary Table 3: Final result of quality control for the whole cohort (n=65) and divided due to availability of complete MRI data for central review. There was no difference in ratio of observed deviations between the subgroups of review with versus without complete MRI data (Chi^2 p=0.875).

MRI – magnetic resonance imaging

Types of deviation and review criteria in pre-treatment central quality control of tumor bed boost in medulloblastoma – An analysis of the German Radiotherapy Quality Control Panel in the SIOP PNET5 MB trial. Strahlentherapie und Radioonkologie. Dietzsch S et al. Department for Radiation Oncology, University of Leipzig Medical Center, Leipzig, Germany. Email: stefan.dietzsch@medizin.uni-leipzig.de
