# Supplementary material for: Types of deviation and review criteria in pretreatment central quality control of tumor bed boost in medulloblastoma—an analysis of the German Radiotherapy Quality Control Panel in the SIOP PNET5 MB trial
Source: Strahlenther Onkol. 2021 Aug 5;198(3):282–90. doi: 10.1007/s00066-021-01822-0 (PMC8863746; doi:10.1007/s00066-021-01822-0)
Supplement: Supplementary file 6 — Supplementary Table 4: Definitions of target volume deviations of other trials [file 66_2021_1822_MOESM6_ESM.pdf]

	Per protocol	Acceptable deviation	Unacceptable deviation
**CATNON trial**			
**GTV**	Hypodense areas on CT (corresponding to T2 or Flair on MRI) included.<br>Plus region of enhancement present on postoperative CT/MRI or the region of enhancement on pre-operative CT/MRI included.<br>Plus tumour resection margin included.	All of the above not fully included. but covered fully by CTV	One or none of the criteria mentioned (T2 area, hypodense area, enchancement)<br>No GTV contoured<br>Not fully contoured/ and potential hypodense areas also not covered by CTV
**CTV**	1.5 - 2 cm volumetric expansion of the GTV is present.<br>Does not extend to contralateral hemisphere when no midline structures like corpus callosum involved.<br>Is not extended into tentorium and meninges by same large margin but a reduced margin of 7-10 mm at these borders	If margin is >1 cm and ≤ 1.4 cm.<br>Extension contralaterally or into skin or into bones without pathologically based evidence or existence of important OAR/sensitive structure within	Margin is < 1 cm<br>Does not cover GTV<br>No CTV present
**PTV**	Defined margin based upon known inaccuracy of set-up, around 5-7 mm.<br>3-D growth algorithm applied (may be reduced towards body border)	Margin less than 3-4 mm	PTV does not cover CTV fully<br>No PTV present
**EORTC 22042–26042**			
**Target Volume Delineation**	PTV covers all CTV and GTV<br>CTV covers all GTV		GTV and CTV outside the PTV; GTV outside the CTV
**GTV to CTV margin**	10 mm	11-15 mm	>15 mm
**EORTC 22033–26033**<br>**(target volume deviations were not predefined, reported were reasons for deviations)**			
**GTV**	Region of high signal intensity on T2 or FLAIR–MRI corresponding to the hypodense area on CT,including any areas of CT enhancement;or operative cavity + any residual tumour	Crosses anatomic boundaries<br>Cannot be judged without MRI<br>incorrect	No GTV<br>2 GTVs
**CTV**	GTV + 1–1.5 cm except at anatomic boundaries where 5 mm is sufficient	Crosses anatomic boundaries<br>Margin incorrect	No CTV; 2 CTVs<br>incorrect
**PTV**	CTV + 3D expansion of 5–7 mm	Margin incorrect; Cannot be judged without CTV	2 PTVs<br>incorrect

Supplementary table 4: Definitions of target volume deviations of other trials[7,8,10]

MRI – magnetic resonance imaging; GTV – gross tumor volume; CTV – clinical target volume; PTV – planning target volume
