# Supplementary material for: Types of deviation and review criteria in pretreatment central quality control of tumor bed boost in medulloblastoma—an analysis of the German Radiotherapy Quality Control Panel in the SIOP PNET5 MB trial
Source: Strahlenther Onkol. 2021 Aug 5;198(3):282–90. doi: 10.1007/s00066-021-01822-0 (PMC8863746; doi:10.1007/s00066-021-01822-0)
Supplement: Supplementary file 7 — Supplementary Figure 3: Example from the case study with two acceptable deviations and mutual compensation of these errors [file 66_2021_1822_MOESM7_ESM.pdf]

# Supplementary Figure 3

A B

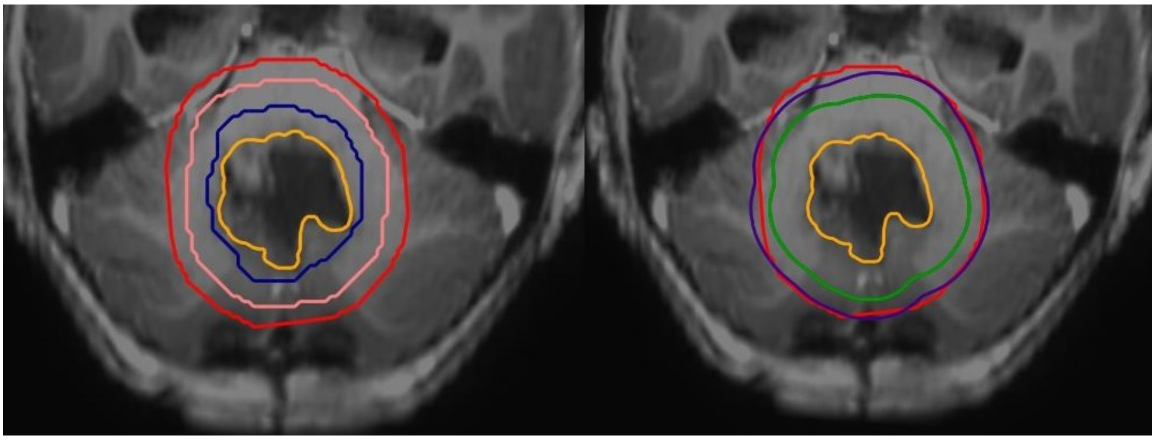

C

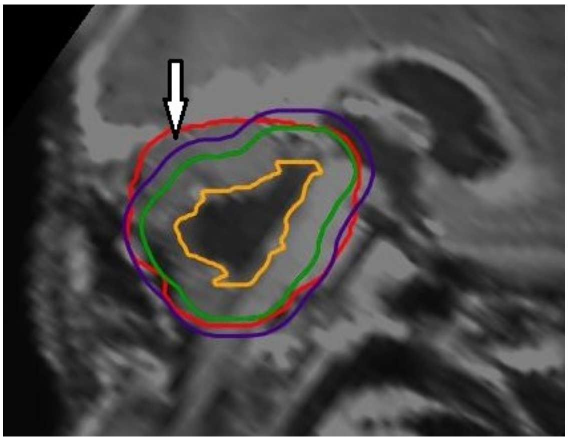

Example two acceptable deviations with mutual compensation of errors

A) the $GTV_{tumorbed}$ of the local radiooncologist (blue) is larger than necessary (reference $GTV_{tumorbed}$ -orange) and includes uninvolved normal brain tissue; the CTV margin (pink) is 5mm and therefore less than recommended

B and C) because of two potential acceptable deviations a reference CTV (green) and PTV (violet) was created; the PTV of the local radiooncologist (red) encompass the reference CTV (green) → acceptable deviation; cranial the PTV of the local radiooncolgist (red) is larger than the reference PTV (violet) but not more than 5mm → acceptable deviation

Types of deviation and review criteria in pre-treatment central quality control of tumor bed boost in medulloblastoma – An analysis of the German Radiotherapy Quality Control Panel in the SIOP PNET5 MB trial. Strahlentherapie und Radioonkologie. Dietzsch S et al. Department for Radiation Oncology, University of Leipzig Medical Center, Leipzig, Germany. Email: stefan.dietzsch@medizin.uni-leipzig.de
